
JOURNAL INFORMATION
==============================
NLM Title Abbreviation: ScientificWorldJournal
Journal ID: ScientificWorldJournal
NLM Journal ID: 101131163
Journal ID: TSWJ

The Scientific World Journal

ISSN: 2356-6140
EISSN: 1537-744X
Publisher: Wiley

ARTICLE INFORMATION
==============================
PMCID: PMC5849222
PMID: 18836666
DOI: 10.1100/tsw.2008.131
Article ID: 350452
Article version: 1
Subjects: Directions in Science

Wound Repair Capability in EDS Fibroblasts can be Retrieved by Exogenous Type V Collagen

Viglio Simona 1
Zoppi Nicoletta 2
Sangalli Antonella 3
Gallanti Angelo 1
Barlati Sergio 2
Mottes Monica 3
Colombi Marina 2
Valli Maurizia 1 *
1 Department of Biochemistry A. Castellani, University of Pavia, 27100 Pavia, Italy
2 Department of Biomedical Sciences and Biotechnology, Division of Biology and Genetics, University of Brescia, 25123 Brescia, Italy
3 Department of Mother and Child, Biology and Genetics, University of Verona, 37134 Verona, Italy
*Maurizia Valli: mauriv@unipv.itAcademic Editor: Martin Goette

Publication date: 2008
Electronic publication date: 2008 Oct 3
Volume: 8
First page: 956
Last page: 958
Received 2008 Jul 9; Revised 2008 Sep 17; Accepted 2008 Sep 21
Copyright: Copyright © 2008 Simona Viglio et al.
Copyright year: 2008
License: This is an open access article distributed under the Creative Commons Attribution License, which permits unrestricted use, distribution, and reproduction in any medium, provided the original work is properly cited.
License URL: https://creativecommons.org/licenses/by/3.0/

Keywords: Ehlers-Danlos syndrome, type V collagen, integrin, heparan sulfate, wound repair

==============================
Directions in Science
*TheScientificWorldJOURNAL* (2008) 8, 956–958
ISSN 1537-744X; DOI 10.1100/tsw.2008.131

TheScientificWorldJOURNAL
www.thescientificworld.com

### Wound Repair Capability in EDS Fibroblasts can be Retrieved by Exogenous Type V Collagen

Simona Viglio[1], Nicoletta Zoppi[2], Antonella Sangalli[3], Angelo Gallanti[1], Sergio Barlati[2], Monica Mottes[3], Marina Colombi[2], and Maurizia Valli[1,*]

*[1]Department of Biochemistry A. Castellani, University of Pavia, 27100 Pavia, Italy; [2]Department of Biomedical Sciences and Biotechnology, Division of Biology and Genetics, University of Brescia, 25123 Brescia, Italy; [3]Department of Mother and Child, Biology and Genetics, University of Verona, 37134 Verona, Italy*

**E-mail:** mauriv@unipv.it

*Received July 9, 2008; Revised September 17, 2008; Accepted September 21, 2008; Published October 3, 2008*

**KEYWORDS:** Ehlers-Danlos syndrome, type V collagen, integrin, heparan sulfate, wound repair

Ehlers-Danlos syndrome (EDS) refers to a group of rare, inherited, connective tissue disorders affecting joints, skin, and blood vessels[1]. The most common type, according to the current clinical classification, is Classical EDS, comprising the former EDS type I (OMIM 130000; http://www.ncbi.nlm.nih.gov/entrez/dispomim.cgi?id=130000) and type II (OMIM 130010; http://www.ncbi.nlm.nih.gov/entrez/dispomim.cgi?id=130010) characterized by joint hypermobility, skin hyperextensibility, and impaired wound healing with abnormal scars. In Classical EDS, mutations affecting type V collagen genes have been found and the most common mutations lead to a nonfunctional COL5A1 allele[1]. Type V collagen, a minor component of extracellular matrix (ECM), is essential for type I collagen fibril formation, acting as nucleator and regulator of fibril diameter[2,3], and plays a fundamental role in the development of functional connective tissues. Mutations in type V collagen caused *in vitro* the deposition of a defective fibrillar network of collagens and fibronectin associated to an altered pattern of integrins[4,5]. Since both integrins and fibronectin have a pivotal role in wound repair[6], the impaired wound healing observed in Classical EDS could therefore be explained by the poor organization of type V collagen–rich connective tissues, such as skin.

On these bases, a very recent paper[7] investigated *in vitro* wound repair in fibroblasts derived from Classical EDS patients with type V collagen deficiency and a markedly delayed wound healing, in order to evaluate their repair capability and their adequacy as tools for testing the effect of potential repairing therapeutics. In this study, fibroblasts deposed, as expected, a defective ECM lacking fibrillar networks of collagens and fibronectin, and showed a down-regulation of $\alpha_5\beta_1$ and $\alpha_2\beta_1$ integrins, binding collagen and fibronectin, respectively, while $\alpha_v\beta_3$ integrin, an auxiliary receptor for fibronectin, was up-regulated. The authors designed an *in vitro* wounding assay where mutant fibroblasts were unable to move into a previously scratched area, which simulates a wound, showing a marked delay in repair (Fig. 1a). It is interesting to note that, immediately after scraping, the border between wound and confluent monolayer was sharp in control fibroblasts, whereas in EDS cells, it was not so evident and cells appeared partly detached far beyond the scraping line. After 48 h, control cells had uniformly migrated into the acellular

*Corresponding author.
©2008 with author.
Published by TheScientificWorld; www.thescientificworld.com

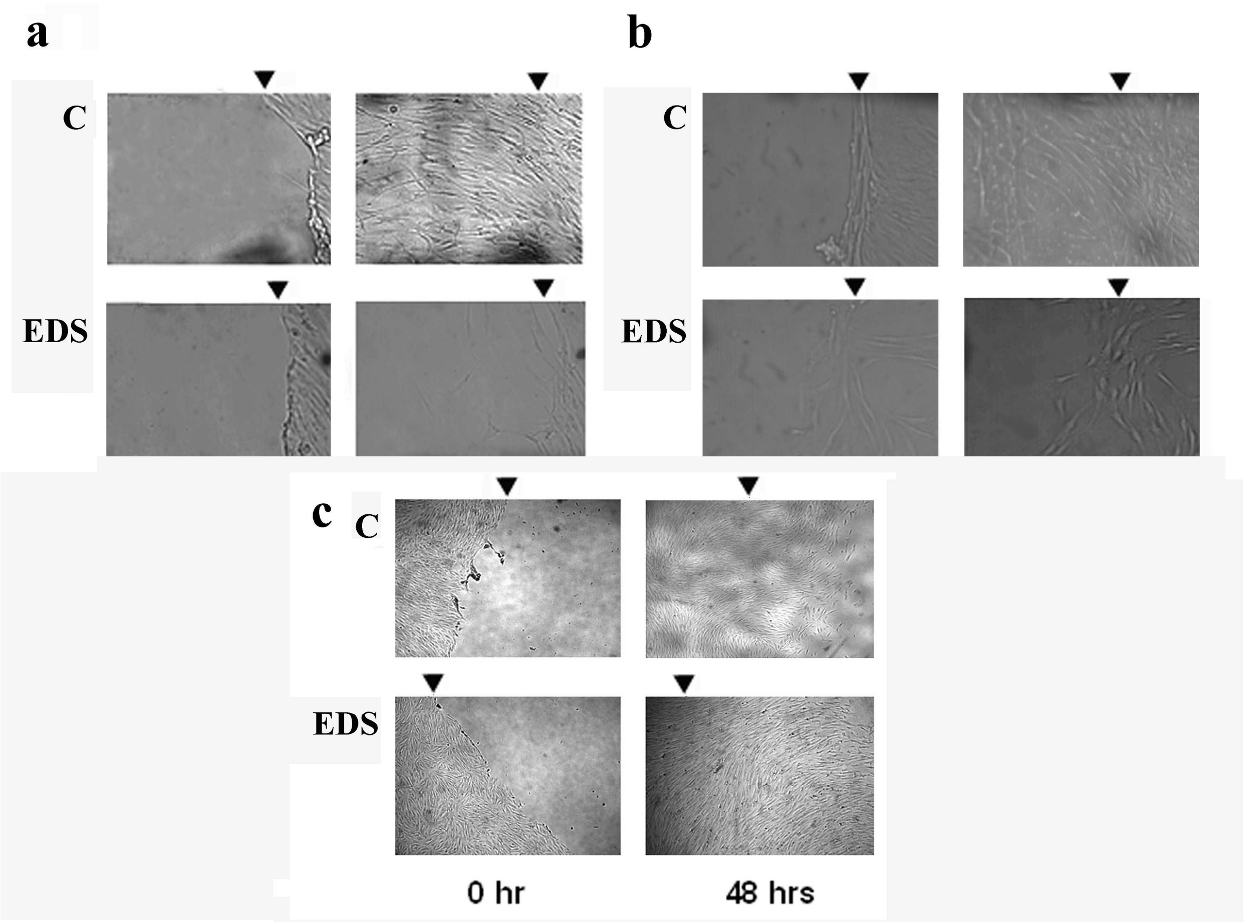

**FIGURE 1.** Wound healing and migration in EDS fibroblasts**.** (a) Fibroblasts of control and Classical EDS patients were scraped (*0 h*) and allowed to migrate for 48 h (*48 h*). (b) Fibroblasts were scraped as above and allowed to migrate in the presence of IGFBP-1. (c) Fibroblasts were grown in medium supplemented with type V collagen. After wounding, fibroblasts were allowed to migrate in the same supplemented medium. Arrowheads indicate the wound edge.

area, whereas EDS cells were still very sparse and even more disorganized than immediately after scraping. In the attempt to improve scratch repair, the authors tested insulin-like growth factor-binding protein-1 (IGFBP-1), known to improve wound healing[8] through signaling via $\alpha_5\beta_1$ integrin, and type V collagen, capable of restoring ECM organization when added to cultured EDS fibroblasts[5]. IGFBP-1 stimulated the migration and scratch repair of control fibroblasts, but not that of EDS strains (Fig. 1b); their abnormal integrin pattern inhibited its positive effect on cell migration. The migratory capability, instead, remarkably improved in the presence of exogenous type V collagen (Fig. 1c); the scraping line in EDS fibroblasts was sharper and they migrated as the controls. The authors concluded that the delayed scrape repair in EDS fibroblasts is caused by the concurrence of impaired fibrillar network and altered integrin pattern. The choice of therapeutics meant for improvement of wound repair in EDS cells should therefore consider the possible involvement of integrins in the cellular response.

Normal wound healing is a highly regulated process and requires several factors. Fibrillar matrix, fibronectin, and its $\alpha_5\beta_1$ integrin receptor allow fibroblast migration and adhesion. Additional ECM molecules, acting as regulators and organizers, are necessary as well for the maintenance of fibrillar matrix functions and integrity. Wound healing impairment can therefore be the consequence of mutations affecting different genes. Murine models, lacking the transmembrane heparan sulfate proteoglycan syndecan-4, a modulator of integrin activity, show significant delayed healing of skin wounds and impaired angiogenesis[9]. In a more recent study[10], it has been demonstrated that *in vitro*–delayed scrape repair in a progeroid form of EDS was associated to a β4GalT-7 gene mutation affecting the biosynthesis of heparan sulfate. Mutations in β4GalT-7 and tenascin-X[11] genes suggest that the extreme clinical heterogeneity in EDS likely derives from genetic heterogeneity. The search for causative mutations must therefore be addressed not only to collagen genes or to genes coding for collagen modifying enzymes, but also to genes coding for nucleators/organizers of the ECM. Complex EDS pathogenesis may be understood only through the knowledge of the fine regulation determining ECM complex architecture.

### REFERENCES

1. Steinmann, B., Royce, P.M., and Superti-Furga, A. (2002) The Ehlers-Danlos syndrome. In *Connective Tissue and Its Inherited Disorders.* Royce, P. and Steimann, B., Eds. Wiley-Liss, New York. pp. 431–523.
2. Kadler, K.E., Hill, A., and Canty-Laird, E.G. (2008) Collagen fibrillogenesis: fibronectin, integrins, and minor collagens as organizers and nucleators. *Curr. Opin. Cell Biol*. **20**, 495–501.
3. Wenstrup, R.J., Florer, J.B., Brunskill, E.W., Bell, S.M., Chervoneva, I., and Birk, D.E. (2004) Type V collagen controls the initiation of collagen fibril assembly. *J. Biol. Chem.* **279**, 53331–53337.
4. Wenstrup, R.J., Florer, J.B., Cole, W.G., Willing, M.C., and Birk, D.E. (2004) Reduced type I collagen utilization: a pathogenic mechanism in COL5A1 haplo-insufficient Ehlers-Danlos syndrome. *J. Cell. Biochem.* **92**, 113–124.
5. Zoppi, N., Gardella, R., De Paepe, A., Barlati, S., and Colombi, M. (2004) Human fibroblasts with mutations in COL5A1 and COL3A1 genes do not organize collagens and fibronectin in the extracellular matrix, down-regulate alpha2beta1 integrin, and recruit alphavbeta3 instead of alpha5beta1 integrin. *J. Biol. Chem.* **279**, 18157–18168.
6. Stupack, D.G. and Cheresh, D.A. (2004) Integrins and angiogenesis. *Curr. Top. Dev. Biol.* **64**, 207–238.
7. Viglio, S., Zoppi, N., Sangalli, A., Gallanti, A., Barlati, S., Mottes, M., Colombi, M., and Valli, M. (2008) Rescue of migratory defects of Ehlers-Danlos syndrome fibroblasts *in vitro* by type V collagen but not insulin-like binding protein-1. *J. Invest. Dermatol.* **128**, 1915–1919.
8. Tsuboi, R., Shi, C.M., Sato, C., Cox, G.N., and Ogawa, H., (1995) Co-administration of insulin-like growth factor (IGF)-I and IGF-binding protein-1 stimulates wound healing in animal models. *J. Invest. Dermatol.* **104**, 199–203.
9. Echtermeyer, F., Streit, M., Wilcox-Adelman, S., Saoncella, S., Denhez, F., Detmar, M., and Goetinck, P. (2001) Delayed wound repair and impaired angiogenesis in mice lacking syndecan-4. *J. Clin. Invest.* **107**, R9–14
10. Götte, M., Spillmann, D., Yip, G.W., Versteeg, E., Echtermeyer, F.G., van Kuppevelt, T.H., and Kiesel, L. (2008) Changes in heparan sulfate are associated with delayed wound repair, altered cell migration, adhesion and contractility in the galactosyltransferase I (beta4GalT-7) deficient form of Ehlers-Danlos syndrome. *Hum. Mol. Genet.* **17**, 996–1009.
11. Schalkwijk, J., Zweers, M.C., Steijlen, P.M., Dean, W.B., Taylor, G., van Vlijmen, I.M., van Haren, B., Miller, W.L., and Bristow, J. (2001). A recessive form of the Ehlers-Danlos syndrome caused by tenascin-X deficiency. *N. Engl. J. Med.* **345**, 1167–1175.

**This article should be cited as follows:**

Viglio, S., Zoppi, N., Sangalli, A., Gallanti, A., Barlati, S., Mottes, M., Colombi, M., and Valli, M. (2008) Wound repair capability in EDS fibroblasts can be retrieved by exogenous type V collagen. *TheScientificWorldJOURNAL* **8**, 956–958. DOI 10.1100/tsw.2008.131.
